# Scaling of morphogenetic patterns in reaction-diffusion systems

**DOI:** 10.1016/j.jtbi.2016.05.035

**Published:** 2016-09-07

**Authors:** Manan’Iarivo Rasolonjanahary, Bakhtier Vasiev

**Affiliations:** Department of Mathematical Sciences, University of Liverpool, Liverpool, UK

**Keywords:** Pattern formation, Developmental biology, Mathematical modelling, Robustness and scaling

## Abstract

Development of multicellular organisms is commonly associated with the response of individual cells to concentrations of chemical substances called morphogens. Concentration fields of morphogens form a basis for biological patterning and ensure its properties including ability to scale with the size of the organism. While mechanisms underlying the formation of morphogen gradients are reasonably well understood, little is known about processes responsible for their scaling. Here, we perform a formal analysis of scaling for chemical patterns forming in continuous systems. We introduce a quantity representing the sensitivity of systems to changes in their size and use it to analyse scaling properties of patterns forming in a few different systems. Particularly, we consider how scaling properties of morphogen gradients forming in diffusion-decay systems depend on boundary conditions and how the scaling can be improved by passive modulation of morphogens or active transport in the system. We also analyse scaling of morphogenetic signal caused by two opposing gradients and consider scaling properties of patterns forming in activator–inhibitor systems. We conclude with a few possible mechanisms which allow scaling of morphogenetic patterns.

## Highlights

• Measure of sensitivity of patterns to change in the medium size is introduced.
• Scaling properties of patterns forming in reaction-diffusion systems are studied.
• Examples of improving of scaling under modulation are shown.
• Scaling in systems with active transport and opposing gradients is analysed.
• Scaling of Turing patterns with and without modulation is studied.

## 1 Introduction

The greatest manifestation of biological development is given by embryogenesis when fully functional multicellular organisms arise from a single fertilised cell. The “elementary” processes underlining embryogenesis are cellular proliferation, differentiation and migration. Cellular differentiation is considered as being most directly related to biological pattern formation and as such was studied in great details. It is known that cells differentiate according to their position and positional information is commonly given by concentrations of biochemical substances which are called “morphogens”. The classical illustration of how development of tissue or organism is conditioned by a concentration of morphogen is given by the French Flag Model (Wolpert, 1969). This model demonstrates how a simple, linear concentration profile of a morphogen can define domains of cellular determination in an otherwise homogeneous tissue. The linear concentration profiles with predefined concentration values on tissue boundaries can form naturally in various settings (Crick, 1970) and an important feature of this profile is that it scales with the size of tissue. Despite its mathematical simplicity, the French Flag Model had a great impact on design of experiments and interpretation of experimental results in developmental biology for many decades (Wolpert, 2011).

Morphogenetic patterns observed in experimental conditions are, as a rule, not linear and exploring and understanding their scaling properties is one of the biggest challenges in contemporary biology. Various scaling mechanisms were recently suggested on the basis of mathematical studies of morphogenetic patterns (Ben-Zvi and Barkai, 2010, Ishihara and Kaneko, 2006, Lo et al., 2015, Umulis and Othmer, 2012). Many of them are based on the effect of so called passive and active modulations (Umulis and Othmer, 2013). These mechanisms involve the chemical (modulator) whose concentration depends on the size of the biological object and who affects the dynamics of morphogen concentration. Passive modulation implies no feedback from the morphogen to the modulator bringing typically to linear modulator's kinetics (Umulis and Othmer, 2013). Active modulation involves the impact of morphogen concentration to the dynamics of modulator which closes the loop for their mutual interactions (Ben-Zvi and Barkai, 2010). Other scaling mechanisms introduced in the literature are based on the alteration of transportation (i.e. so called “shuttling” mechanism (Ben-Zvi et al., 2008)) or on the impact of transition processes to the differentiation of cells (i.e. so called pre-steady state patterns (Bergmann et al., 2007)).

Theoretical studies of scaling mechanisms are mainly focused on processes which allow perfect scaling while in experimental conditions scaling can be achieved/verified only with certain accuracy. For example, the concentration profile of Bicoid in the fly embryo can be approximated by an exponential function with the characteristic length of about 1/3 of the size of the embryo. It is shown that this ratio varies within 10% for embryos whose sizes differ more than four times (Gregor et al., 2007a). So, for the given example the question to ask is whether existing models describing formation of Bicoid profile allow scaling with the observed precision. This leads to more general question of what mechanisms allow the scaling with a given precision rather than what mechanisms allow perfect scaling.

To address the above questions, one would need to operate with a quantity describing the precision of scaling. In the next section, we introduce a quantity called “sensitivity factor” which will let us to define sensitivity of the morphogenetic patterns to the changes in size of the system and therefore to estimate how good scaling is. Then, we analyse patterns forming in diffusion-decay systems and study their scaling properties in systems with and without modulation as well as with and without active transport. We conclude this work with analysis of scaling properties of patterns forming in activator–inhibitor systems.

### 1.1 Definition of sensitivity factor

**Fig. 1:**
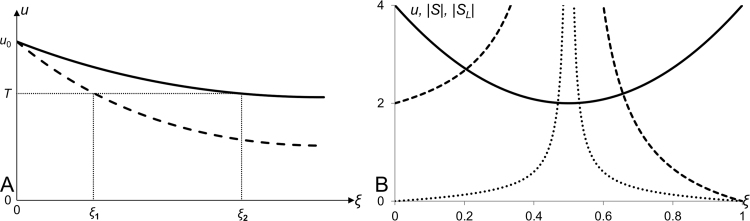
Introducing sensitivity factor for morphogen gradient. A: Sample morphogen profiles for two different medium sizes (*L*_1_ and *L*_2_) are presented as functions of relative position (*ξ*=*x*/*L*). The relative positions *ξ*_1_ and *ξ*_2_ are locations of the points with the same value of *u* (*u*=*T*) for the two profiles. B: Plots of sample symmetric profile (represented by the quadratic function *u*=0.5*ξL*^2^(*ξ*−1)+4, solid line) and absolute values of its sensitivity factor, *S*, (defined by the Eq. (1), dotted line) and scaling coefficient, *S_L_*, (defined by the Eq. (2), dashed line). Both, *S* and *S_L_*, tend to infinity at *ξ*=0.5 and equal to zero at *ξ*=1. The obvious difference between *S* and *S_L_* is that *S* is symmetric (as expected for the symmetric profile) while *S_L_* is not. Thus, the sensitivity factor defined by the Eq. (1) is an improvement of the scaling coefficient defined by the Eq. (2). As the quality of scaling is rather defined by the magnitude of *S* (or *S_L_*) we plot absolute values of these quantities (also in Fig. 2 to Fig. 5) as this serves better for the illustrative purposes.

In order to analyse scaling properties of morphogenetic gradients, we need to introduce a formal definition of scaling precision. For this purpose, we consider (hypothetical) morphogenetic profiles occurring in two objects of different sizes, say *L*_1_ and *L*_2_. These profiles can be described by functions *u*(*ξ*, *L*_1_) and *u*(*ξ*, *L*_2_), where *ξ*=*x*/*L* represents a coordinate relative to the medium size and varies in the range [0,1] for both objects. We note that if the profile is scaled across the two objects then *u*(*ξ*, *L*_1_)=*u*(*ξ*, *L*_2_) for any point *ξ*. Generally, this is not true and from *u*(*ξ*_1_, *L*_1_)=*u*(*ξ*_2_, *L*_2_), it does not follow that *ξ*_1_=*ξ*_2_ (see Fig. 1A). To quantify the deformation of the pattern in response to the changes in the medium size, let us consider small variations in *L* and in *ξ* for which we can use the linear approximation: $u\left(\xi_{2},L_{2}\right)=u\left(\xi_{1},L_{1}\right)+u_{\xi }^{\prime }\left(\xi_{2}-\xi_{1}\right)+u_{L}^{\prime }\left(L_{2}-L_{1}\right)$. Assuming that *u*(*ξ*_1_, *L*_1_)=*u*(*ξ*_2_, *L*_2_) we get $\xi_{2}-\xi_{1}=-\frac{u_{L}^{\prime }}{u_{\xi }^{\prime }}\left(L_{2}-L_{1}\right),$$\xi_{2}-\xi_{1}=-\frac{{u\prime }_{L}}{{u\prime }_{\xi }}\left(L_{2}-L_{1}\right)$, i.e. the deformation of the profile is proportional to the change in the size of the system with the coefficient of proportionality representing the local sensitivity and named “sensitivity factor”:

(1) $$S=-\left(\frac{\partial u}{\partial L}\right){\left(\frac{\partial u}{\partial \xi }\right)}^{-1}$$

Thus the sensitivity factor, *S*, is a function of the length of the medium, *L*, and the relative position, *ξ.* It defines a rate of shift of a point with a given level of morphogen concentration, *u*, when the medium size is varied; positive values correspond to the shift towards the right border of the medium and negative values – to the left. In the case of perfect scaling, there is no such shift, i.e. *S*(*ξ*, *L*)≡0. Good alternative to the definition (1) is given by the product *S*_1_=S*L which defines the deformation of pattern, Δ*ξ*, in terms of the relative change in medium size, that is: $\Delta \xi =S_{1}\frac{\Delta L}{L}$. However in the following, we will use the definition given by the Eq. (1).

The definition of the sensitivity factor given by the Eq. (1) can be seen as a correction of the one introduced earlier (de Lachapelle and Bergmann, 2010) and given as:

(2) $$S_{L}=-\left(\frac{\partial u}{\partial L}\right){\left(\frac{\partial u}{\partial x}\right)}^{-1}\frac{L}{x}.$$

As compared to the sensitivity factor given by the Eq. (1) the quantity defined by Eq. (2) (named “scaling coefficient”) has a few drawbacks introduced by its explicit dependence on coordinate, *x*. Particularly, the scaling coefficient defined by (2) is sensitive to the choice of border from which the coordinate is measured and asymmetric for symmetric profiles. Indeed, for a symmetric pattern the partial derivatives $\frac{\partial u}{\partial L}$ and $\frac{\partial u}{\partial x}$ are also symmetric (or antisymmetric), that is, they have the same magnitudes in equidistant points on two sides of symmetric pattern (although can differ by sign). At the same time, the factor $\frac{L}{x}$ is not symmetric and introduces asymmetry in “should be” symmetric scenario. This effect is illustrated by panel B in Fig. 1 where we can see that for symmetric profile the quantity *S* defined by Eq. (1) is symmetric while the quantity *S*_*L*_ defined by Eq. (2) is not.

### 1.2 Diffusion-decay systems

According to experimental observations, morphogen gradients often have an exponential shape. The typical example is given by the transcriptional factor Bicoid in the early embryo of the fruit fly *Drosophila melanogaster* (Driever and Nussleinvolhard, 1988, Gregor et al., 2008). Exponential profile occurs as a solution in mathematical models describing the dynamics of morphogen under the assumption that it diffuses (with a constant rate) and degrades (linearly), i.e. in so called “diffusion-decay” models (Lander et al., 2002). The concentration of morphogen can be fixed on the boundaries of the tissue (i.e. the morphogen passively diffuses from one boundary to another) or there is no flux on the boundaries and the morphogen is produced in some area inside the domain. These assumptions reflect common settings in biological objects. For example, the maternal Bicoid mRNA in *D. melanogaster* embryo is localised in a small region on its apical side, the Bicoid protein is produced in this region, diffusively spreads along the entire embryo and decays (Gregor et al., 2008). Assuming that the area of protein production is small as compared to the total size, *L*, of the embryo, the dynamics of the morphogen can be described by the diffusion-decay equation:

(3) $$\frac{\partial u}{\partial t}=D_{u}\frac{\partial^{2}u}{\partial x^{2}}-k_{u}u$$

with Neumann boundary conditions:

(4) $${\frac{\partial u}{\partial x}|}_{x=0}=-q\;\mathrm{and}{\frac{\partial u}{\partial x}|}_{x=L}=0.$$

Here, terms on the right hand side of (3) stand for diffusion (*D*_*u*_ is the diffusion coefficient) and decay (*k*_*u*_ is the decay rate) and non-zero flux, *q*, at *x*=0 in (4) replaces the production of Bicoid protein on the anterior side of the embryo. The stationary solution of Eq. (3) with boundary conditions (4) is given by the superposition of the exponents:

(5) $$u\left(x\right)=q\lambda \frac{\exp\left(\frac{x-L}{\lambda }\right)+\exp\left(\frac{L-x}{\lambda }\right)}{\exp\left(\frac{L}{\lambda }\right)-\exp\left(\frac{-L}{\lambda }\right)}$$

which, for sufficiently large *L* (*L*>>*λ*), can be approximated by a single exponent *u*(*x*)=*qλ*exp(−*x*/*λ*). Here, $\lambda =\sqrt{D_{u}/k_{u}}$gives a characteristic length of the profile.

Eq. (3) is often considered under Dirichlet boundary conditions in which case the stationary solution can also be easily defined. For example, if *u*(*x*=0)=*u*_0_ and *u*(*x*=*L*)=0 the stationary solution is given as:

(6) $$u\left(x\right)=u_{0}\frac{\exp\left(\frac{L-x}{\lambda }\right)-\exp\left(\frac{x-L}{\lambda }\right)}{\exp\left(\frac{L}{\lambda }\right)-\exp\left(\frac{-L}{\lambda }\right)}$$

where *u*_0_ is the boundary value at *x*=0 and *λ* has the same definition as above. This solution, for sufficiently large *L* (*L*>>*λ*), can be approximated by a single exponent *u*(*x*)=*u*_0_exp(−*x*/*λ*).

### 1.3 Scaling in diffusion-decay systems

**Fig. 2:**
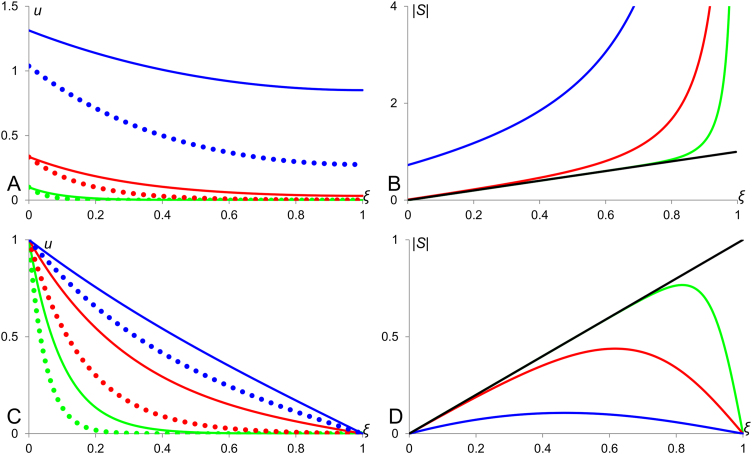
Scaling in diffusion-decay systems. Panels A and C show plots of stationary profiles forming under Neumann (panel A) and Dirichlet (panel C) boundary conditions while panels B and D – plots of corresponding sensitivity factors. In all panels, green, red and blue lines correspond to *λ*=1/10, 1/3 and 1 respectively. Black lines in panels B and D show plot of sensitivity factor for stationary profile approximated by a single exponent, i.e. *u*(*ξ*)=*qλ*exp(−*ξL*/*λ*) or *u*(*ξ*)=*u*_0_exp(−*ξL*/*λ*) both having identical sensitivity factors.

Now, we can analyse how the scaling properties of diffusion-decay system depend on its parameters and boundary conditions. Stationary solution (5) (of the system (3) with Neumann boundary conditions (4)) for three different values of the ratio *λ*/*L* are plotted in Fig. 2A with corresponding sensitivity factors shown in Fig. 2B. We can see that the profiles for lower value of *λ*/*L* scale better than those corresponding to higher value of this ratio. Similarly, stationary concentration profiles given by Eq. (6) (that is, solutions of the diffusion-decay system (3) under the Dirichlet boundary conditions (*u*(*x*=0)=1; *u*(*x*=*L*)=0)) for three different values of the ratio *λ*/*L* are shown in Fig. 2C with corresponding sensitivity factors given in Fig. 2D. Here, we see the opposite scenario – the sensitivity factor decreases when the characteristic length is increasing. In the limit case, when the ratio *λ*/*L* tends to infinity, the profile becomes linear (transfers into the profile in the French Flag model) and its sensitivity factor drops to zero. The sensitivity factors for the solutions (5) and (6) are analytically represented by formulas

(7) $$S=-\frac{\left(\xi -2\right)\left(e^{\frac{\xi L}{\lambda }}+e^{-\frac{\xi L}{\lambda }}\right)-\xi \left(e^{\frac{L\left(\xi -2\right)}{\lambda }}+e^{-\frac{L\left(\xi -2\right)}{\lambda }}\right)}{L\left(e^{\frac{L}{\lambda }}-e^{-\frac{L}{\lambda }}\right)\left(e^{\frac{L\left(\xi -1\right)}{\lambda }}-e^{-\frac{L\left(\xi -1\right)}{\lambda }}\right)}$$

and

(8) $$S=-\frac{\left(\xi -2\right)\left(e^{\frac{\xi L}{\lambda }}-e^{-\frac{\xi L}{\lambda }}\right)+\xi \left(e^{-\frac{L\left(\xi -2\right)}{\lambda }}-e^{\frac{L\left(\xi -2\right)}{\lambda }}\right)}{L\left(e^{\frac{L}{\lambda }}-e^{-\frac{L}{\lambda }}\right)\left(e^{\frac{L\left(\xi -1\right)}{\lambda }}+e^{-\frac{L\left(\xi -1\right)}{\lambda }}\right)}$$

respectively. Samples of profiles presented in Panels B and D of Fig. 2 are plotted according to these formulas. Comparing sensitivity factors given by formulas (7) and (8) one can show that the system under Dirichlet boundary condition scales always better than the one under the Neumann boundary conditions. Note that when the characteristic length *λ* gets larger the absolute value of sensitivity factor is decreasing in the case of the Dirichlet boundary condition and increasing in the case of the Neumann boundary conditions. One can show, using linear approximation of exponential terms that when *λ* tends to infinity, *S* in Eq. (8) tends to zero (the morphogen profile gets linear like in French Flag Model and its scaling becomes perfect). In the opposite case when *λ* tends to zero, sensitivity factor under Neumann conditions decreases and under Dirichlet condition increases and they merge at line *S*=*−ξ*/*L* except for the edge where *ξ*=1, that is:

$$\underset{\lambda \to 0}{\left|S\right|}\to \{\begin{array}{c} \frac{\xi }{L}\;\mathrm{for}\;\xi <1\\ \{\begin{array}{c} 0\;\mathrm{for}\;\mathrm{Dirichlet}\;\mathrm{BC}\\ \infty \;\mathrm{for}\;\mathrm{Neumann}\;\mathrm{BC} \end{array}\mathrm{for}\;\xi =1 \end{array}.$$

The line *S*=*−ξ*/*L* which limits sensitivity factors for Neumann and Dirichlet boundary conditions also represents the sensitivity factor for the single exponent approximations *u*(*ξ*)=*qλ*exp(−*ξL*/*λ*) (Neumann boundary conditions) and *u*(*ξ*)=*u*_0_exp(−*ξL*/*λ*) (Dirichlet boundary conditions). This line is shown in panels B and D of Fig. 2.

### 1.4 Scaling in a diffusion-decay system with modulator

The above results indicate that scaling properties of patterns forming in diffusion-decay systems are different at different locations and depend on their characteristic length and boundary conditions. Although the scaling is better under the Dirichlet boundary conditions (as compared to the mixed and Neumann boundary conditions), it is still not good enough: for example, the sensitivity factor, *S*=−0.41, in the centre (*ξ*=0.5) of the system with the relative characteristic length *λ*/*L*=1/3, which means that the deformation of the pattern corresponding to 10% shift from the middle of the medium will be achieved when the size of the medium is changed from *L*_1_=1 to *L*_2_=1.25. As compared with experimental observations (for example on the mentioned above Bicoid profile in fly embryo (Gregor et al., 2008)) this cannot be considered satisfactory. In order to improve scaling properties of morphogen gradient, one needs to use more sophisticated models. Such models, with an extra variable, which is called modulator have been proposed by many researchers (Umulis and Othmer, 2013) with various kinds of postulated relationships between morphogen and modulator (see for example the expansion-repression mechanism in (Ben-Zvi and Barkai, 2010; Ben-Zvi et al., 2011)). One of the simplest ideas of modulation (Ishihara and Kaneko, 2006), can be derived from the observation made on diffusion-decay systems under the Neumann boundary conditions (Fig. 2A). We note that the solution (5) levels off (gets less dependent on the coordinate) with an increase of the relative characteristic length, *λ*/*L*. This solution can be approximated as *u*≈*qλ*^2^/*L* when *λ*/*L*>>1 and thus can be used as a measure of the medium size. Therefore, we can consider the substrate, with levelled off profile, as a modulator for another variable which represents a scaled morphogen. For example, we can assume that the modulator (*u*) as described by the Eq. (3) affects the decay rate of the morphogen (*v*) whose stationary profile is given by the diffusion-decay equation under the mixed boundary conditions:

(9) $$D_{v}\frac{d^{2}v}{dx^{2}}-k_{v}vu^{n}=0;\;v\left(x=0\right)=1;\;\;{\frac{dv}{dx}|}_{x=L}=0.$$

where parameter *n* defines the strength of the modulator's influence to the morphogen decay. The boundary conditions in (9) can be justified by referencing to the following settings. The tissue is isolated (this gives zero flux boundary condition at *x*=*L*) and there is a production of the morphogen in a small area located in the vicinity of the medium edge at *x*=0. If the production is nonlinear then the concentration of morphogen can stabilise at a certain level, called stable stationary state, and thus we can assume that *v*=1 is the concentration corresponding to steady state of the morphogen kinetics in the production area.

The solution of Eq. (9) is given by superposition of two exponents (in the assumption that concentration, *u*, of the modulator is constant throughout the tissue). However, if the size of the medium is large enough, then *v*(*x*) can be approximated by a single exponent, which is in the case of *n*=2 given by the formula:

(10) $$v\left(x\right)\approx v_{0}e^{-x\sqrt{\frac{k_{v}u^{2}}{D_{v}}}}=v_{0}e^{-xu\sqrt{\frac{k_{v}}{D_{v}}}}=v_{0}e^{-x\frac{D_{u}q}{k_{u}L}\sqrt{\frac{k_{v}}{D_{v}}}}=v_{0}e^{-\xi \frac{D_{u}q}{k_{u}}\sqrt{\frac{k_{v}}{D_{v}}}}.$$

The morphogen profile given by solution (10) is a function of the relative position, *ξ*=*x*/*L*, rather than the position *x* and therefore its sensitivity factor is identically zero, i.e. for *n*=2 it scales perfectly. In the case of *n=*0 the approximation of concentration profile is given by the exponent:

(11) $$v\left(x\right)\approx v_{0}e^{-x\sqrt{\frac{k_{v}}{D_{v}}}}=v_{0}e^{-\xi L\sqrt{\frac{k_{v}}{D_{v}}}}$$

which is a function of both the relative position *ξ* and the size *L*. This case corresponds to the model without modulator and the profile given by (11) has scaling properties similar to those shown in Fig. 2. An approximation of concentration profile in the case when *n*=1 is given by the formula:

(12) $$v\left(x\right)\approx v_{0}e^{-x\sqrt{\frac{k_{v}u}{D_{v}}}}=v_{0}e^{-x\sqrt{\frac{D_{u}q}{k_{u}L}\frac{k_{v}}{D_{v}}}}=v_{0}e^{-\frac{x}{\sqrt{L}}\sqrt{\frac{D_{u}q}{k_{u}}\frac{k_{v}}{D_{v}}}}=v_{0}e^{-\xi \sqrt{L}\sqrt{\frac{D_{u}q}{k_{u}}\frac{k_{v}}{D_{v}}}}.$$

**Fig. 3:**
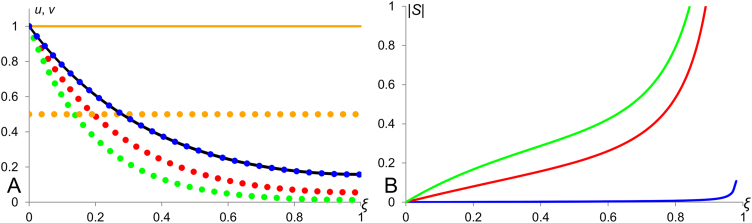
Scaling in a diffusion-decay system with modulation. Morphogen profiles and their sensitivity factors in the system (3, 9) with *n*=0, 1 and 2 in Eq. (9). A: The black line corresponds to *v*-profile when *L*=1 for all three cases. All the dotted lines stand for *L*=2 and solid lines for *L*=1. The green, red and blue colours correspond to *n*=0, 1 and 2 respectively. Orange lines represent profiles of the modulator (given by the Eq. (3)). B: Plots of absolute value of sensitivity factors: the green, red and blue colours correspond to *n*=0, 1 and 2 respectively. The blue profile shows the lowest sensitivity corresponding to the best scaling. The values of model parameters: *D_u_*=1000, *D_v_*=1, *k_u_*=1, *k_v_*=6.4 and *q*=0.001.

This corresponds to the case of mild modulation; scaling is better than in the case of *n*=0 but still not perfect. All three profiles corresponding to considered values of parameter *n* in the Eq. (9) are shown in Fig. 3. The sensitivity factor for solutions (10) as calculated using the definition (1) is $S_{1}=0$, for (11) it is $S_{2}=-\frac{\xi }{L}$ and for (12) – $S_{3}=-\frac{\xi }{2L}$. We see that the profile (10) scales perfectly as its sensitivity factor is identically zero. The absolute value of *S*_2_ is the largest out of three and thus the scaling properties of profile (11) (diffusion-decay without modulation) is the worse as compared with those of profiles (10) and (12).

Eq. (3) can be modified in a way such that the level of modulator is proportional rather than inverse proportional to the size of the medium (and therefore *n*=−2 in Eq. (9) would lead to perfectly scaling morphogen gradient). This would correspond to an assumption than the modulator is produced everywhere in the tissue but is degraded only in a small area of a fixed size, *a*. Assuming that the degradation area is located in the middle of the medium, the Eq. (3) can be replaced by the following:

(13) $$\frac{\partial u}{\partial t}=D_{u}\frac{\partial^{2}u}{\partial x^{2}}-k\left(x\right)u+p,\;k\left(x\right)=\{\begin{array}{cc} k_{u} & for\;\;\frac{L-a}{2}<x<\frac{L+a}{2}\\ 0 & otherwise \end{array}$$

with zero flux boundary conditions at both ends ${\frac{\partial u}{\partial x}|}_{x=0}={\frac{\partial u}{\partial x}|}_{x=L}=0$, *p* gives the production rate and *k*_*u*_ represents the decay rate. Similarly to the solution of Eq. (3), the solution of (13) levels off with an increase of its relative characteristic length, *λ*/*L*, and can be approximated as *u*≈*pL*/*ak*_*u*_ and thus is proportional to the medium size.

Note that it is important that the Eq. (9), describing the dynamics of morphogen, *v*, is taken under mixed boundary condition: systems (9), (3) or (9), (13) would not allow perfect scaling of morphogen, *v*, at any value of *n* in (9) if Neumann or Dirichlet boundary conditions are applied. Also note, that the system (9), (3) with *n*=2 reproduces the nuclear trapping model (Coppey et al., 2007) in the limit case when the decay of morphogen (Bicoid) takes place only in nuclei and the morphogen profile shows perfect scaling.

### 1.5 Scaling in a system with active transport

To consider the effect of active transport on scaling properties of morphogen gradient we will analyse solutions of the equation:

(14) $$\begin{array}{ccc} D\frac{d^{2}u}{dx^{2}}+c\frac{du}{dx}-ku=0; & u\left(x=0\right)=1; & {\frac{du}{dx}|}_{x=L}=0. \end{array}$$

which is the Eq. (3) extended by the advection term and considered under the mixed boundary conditions. Here, *D* is the diffusion coefficient, *k* is the decay rate and *c* defines the strength of advection. Biological interpretation of the Eq. (14) can be made by referring to possible mechanisms of active molecular transportation. For example, in the case of the Bicoid dynamics in fly embryo (Gregor et al., 2007b, Jaeger, 2009) we can assume that the nuclei in syncytium of oocyte are connected by microtubules which can enforce active Bicoid transportation predominantly oriented in the anterior-to-posterior (or opposite) direction. The Eq. (14) can be analysed under different boundary conditions, however here we consider the case of mixed boundary conditions to keep resemblance with the Bicoid dynamics, which is produced on the anterior side of the embryo (which, in the case of nonlinear production, can be associated with the constant level on the anterior edge (Gregor et al., 2008) and moves and degrades throughout the entire embryo).

The solution of the Eq. (14) is represented by a superposition of two exponents which, in the case of sufficiently large medium, can be approximated by the single exponent:

(15) $$u\approx e^{-\xi \left(\alpha +\sqrt{\alpha^{2}+\beta }\right)},$$

where *α*=*cL*/2*D* counts for the effect of the active transportation and *β*=*kL*^2^/*D* – for that of the morphogen degradation. Looking at these two terms we can conclude that the solution (15) should have scaling properties similar to those of the solution (11). This leads to the problem of what additional conditions would let to improve its scaling properties. To focus on impact of the advection we assume that *β*<<*α*, which, for example, could correspond to the case when the rate of decay, given by parameter *k*, is very low. In this case the solution (15) reduces to $u\approx e^{-\xi \frac{cL}{D}}$ when *c<*0, or *u≈*const when *c>*0. We can see that the perfect scaling can be achieved only if *c* is negative (active flow is towards *ξ*=0 and opposite to the direction of diffusion) and inversely proportional to the medium size *L*, that is *c*=*c*(*L*)=*c*_0_/*L* where *c*_0_ is constant.

**Fig. 4:**
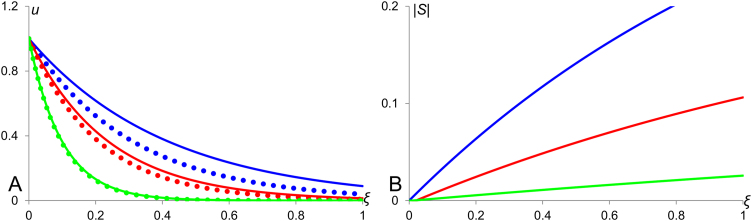
Scaling in a diffusion-advection-decay system. Concentration profiles and their sensitivity factors are shown for the diffusion-advection-decay system (14). A: Continuous and dotted lines correspond to medium sizes *L*=1 and *L*=2 respectively. The blue, red and green colours used to indicate profiles for *α*=1, 2 and 5 respectively. B: Plots of absolute value of sensitivity factors for profiles in panel A using the matching colours are presented. One can see that the scaling is improving with an increase of *α*, that is with an increase of the active flow. Other model parameters: *D*=*k*=1.

In case of the fly embryo, the line of arguments linking the rate of active transportation with the embryo size could be the following. Convective flow is proportional to the density of microtubules which is, in turn, proportional to the number of nuclei. Accepting the argument about the conserved number of nuclei in different sized embryos (Umulis et al., 2008, Umulis, 2009) we can state that the density of nuclei, as considered in anterior-posterior direction, is inversely proportional to the size of the embryo: *ρ*=*N*/*L*. Thus, given the model assumptions, the solution (15), where the factor *α* doesn’t depend on the medium size, *L*, can describe the Bicoid profile in the fly embryo. It scales with the size of embryo provided that the active Bicoid transportation is directed towards the anterior end of the embryo (*c<*0) and the rate of transportation is high as compared with the rate of Bicoid degradation. Plots of solutions (15) together with plots of their sensitivity factors for different values of *α* are shown in Fig. 4.

### 1.6 Scaling in the annihilation model

Differentiation of cells can be affected by more than one morphogen. Besides, while some morphogens promote the differentiation others can inhibit it. The problem of scaling in such systems is not necessarily reduced to the scaled gradients of each of involved morphogens. In this section we will consider a simple example of the system involving two interacting morphogens. Again, we can refer to the development of the fly embryo which can be considered as initiated by gradients of Bicoid and Nanos. These two morphogens are expressed in the opposite sides of the embryo and may be considered as mutually affecting their diffusion and/or degradation rate (Jaeger, 2009). Thus, their dynamics can be described by the annihilation model (Lo et al., 2015, Maini et al., 2012) which is represented as:

(16) $$\{\begin{array}{c} \frac{\partial u}{\partial t}=D_{u}\frac{\partial^{2}u}{\partial x^{2}}-k_{u}uv,\\ \frac{\partial v}{\partial t}=D_{v}\frac{\partial^{2}v}{\partial x^{2}}-k_{v}uv, \end{array}$$

where *D*_*u*_ and *D*_*v*_ are the diffusion coefficients and *k*_*u*_ and *k*_*v*_ specify the decay rates of variables *u* and *v*, which can be considered as representing Bicoid and Nanos respectively. The opposing gradients of *u* and *v* can form in the system (16) under the Dirichlet boundary conditions when nonzero values of the variables *u* and *v* are set at the opposite boundaries.

Let us assume that the morphogen *u* promotes the transcription of certain gene while *v* inhibits it so that the transcription (or differentiation) signal, *w*, is given by the linear combination of *u* and *v*: *w*(*x*, *L*)=*β*_1_*u*(*x*, *L*)−*β*_2_*v*(*x*, *L*) with positive constants *β*_1_ and *β*_2_. Below we show that for the system (16) it is possible to find *β*_1_ and *β*_2_ (or, more generally, the ratio *β*_1_/*β*_2_) such that the signal *w* scales perfectly, that is *w*(*x*, *L*)=*β*_1_*u*(*x*, *L*)−*β*_2_*v*(*x*, *L*)=*w*(*x*/*L*)=*w*(*ξ*) depends only on the relative position. Indeed let us multiply the first equation in (16) by *k*_*u*_ and subtract from it the second multiplied by *k*_*v*_. We get:

$$D_{u}k_{v}\frac{d^{2}u}{dx^{2}}-D_{v}k_{u}\frac{d^{2}v}{dx^{2}}=0$$

Thus for *w*=*D_u_k_v_u*−*D_v_k_u_v* we have:

(17) $$\frac{d^{2}w}{dx^{2}}=0$$

If the opposing gradients in the system (16) are given by the Dirichlet boundary conditions

$$\begin{array}{cc} u\left(x=0\right)=u_{0}, & u\left(x=L\right)=0,\\ v\left(x=0\right)=0, & v\left(x=L\right)=v_{0} \end{array}$$

then the solution of (17) is:

$$w=D_{u}k_{v}u_{0}-\left(D_{u}k_{v}u_{0}+D_{v}k_{u}v_{0}\right)\frac{x}{L}$$

which is a function of the relative position *x*/*L*, *w*=*w*(*x*/*L*) and therefore scales with the size of the medium. At the same time the stationary solutions of *u* and *v* in the system (16) (see derivations in (Ben-Naim, 1992)) do not scale with the medium size.

**Fig. 5:**
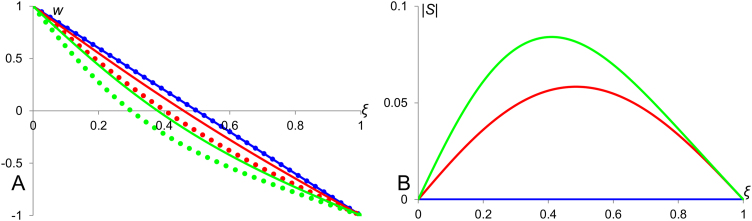
Scaling in the annihilation model. Plots of w-profiles and their sensitivity factors in the annihilation model (16). A: Continuous and dotted lines are used for plotting w-profiles for medium sizes *L*=1 and *L*=2 respectively. The blue, red and green colours are used to indicate w-profiles when *k_u_*=1, 5 and 20 respectively. B: Plots of absolute value of sensitivity factors for profiles in panel A using the matching colours are shown. Values of other model parameters: *D_u_*=*D*_v_=*k_v_*=*u*_0_= *v*_0_=1. We can see that the scaling of w-profile improves with the value of *k_u_* getting closer to that of *k_v_*.

The signal, *w*, which is given by the difference *w*=*u−v* scales perfectly for the case of symmetric system (16) when *D*_*u*_=*D*_*v*_ and *k*_*u*_=*k*_*v*_. This is illustrated by Fig. 5 where the plots of w-profiles and their sensitivity factors for three sets of model parameters, which differ only by the value of *k*_*u*_, are shown. We can see that the scaling of *w* is perfect for the symmetric system and gets worse when the difference between *k*_*u*_ and *k*_*v*_ increases, that is, when the system (16) gets more asymmetric. This statement is also confirmed by simulations (not shown) with varied difference between diffusion coefficients *D*_*u*_ and *D*_*v*_.

### 1.7 Scaling of Turing pattern in a system with modulation

In this section, we will analyse scaling properties of morphogenetic patterns forming in the activator-inhibitor systems. The effect of passive modulation on Turing patterns has previously been studied and it was shown that it can result in conservation of number of spikes in media of different sizes and thus to scaling of pattern (Ishihara and Kaneko, 2006). Indeed, the levelled off modulator (like the one in systems (3, 9) and (9, 13)) can be incorporated into other models, including those which show formation of Turing patterns. Turing patterns form in a system of interacting morphogens which is, in the simplest case of two morphogens having linear kinetics, described by the following system:

(18) $$\{\begin{array}{cc} \frac{\partial v}{\partial t}=D_{v}\frac{\partial^{2}v}{\partial x^{2}}+av+bz, & \frac{\partial v}{\partial x}=0\;\;\mathrm{for}\;x=0,L;\\ \frac{\partial z}{\partial t}=D_{z}\frac{\partial^{2}z}{\partial x^{2}}+cv+dz, & \frac{\partial z}{\partial x}=0\;\;\mathrm{for}\;x=0,L. \end{array}$$

This system has a uniform stationary solution (*v*=*z*=0) which, for certain sets of model parameters, is stable when *D*_*v*_ is small and gets unstable and transforms into periodic pattern when *D*_*v*_ is large. This transition is referred as the Turing instability (Vasieva et al., 2013) and the special periodicity of forming patterns can vary in a certain range defined by the values of parameters in the system (18) (Murray, 1982). The drawback of the linear system (18) is that its variables can have both signs and evolve towards infinity under Turing instability. To keep variables positive and bounded (while avoiding references to specific biological models such as those in (Meinhardt and Gierer, 2000, Vasiev, 2004)) we have modified the system (18) by shifting the equilibrium up from (0, 0) into the point (*v*_0_, *z*_0_) in the first quadrant (keeping variables away from the negative values) and added cubic nonlinearity, with coefficient *α*_*v*_, to the “activator” kinetics which leads to the formation of two attractors (in the first quadrant) and thus lets to keep periodic solutions bounded. Modified system which is extended by Eq. (1) for incorporating passive modulation is:

(19) $$\begin{array}{cc} \frac{\partial v}{\partial t}=D_{v}\frac{\partial^{2}v}{\partial x^{2}}+\left(a\left(v-v_{0}\right)+b\left(z-z_{0}\right)-\alpha_{v}{\left(v-v_{0}\right)}^{3}\right)u^{n}, & {\frac{\partial v}{\partial x}|}_{x=0}={\frac{\partial v}{\partial x}|}_{x=L}=0;\\ \frac{\partial z}{\partial t}=D_{z}\frac{\partial^{2}z}{\partial x^{2}}+\left(c\left(v-v_{0}\right)+d\left(z-z_{0}\right)\right)u^{n}, & {\frac{\partial z}{\partial x}|}_{x=0}={\frac{\partial z}{\partial x}|}_{x=L}=0. \end{array}$$

**Fig. 6:**
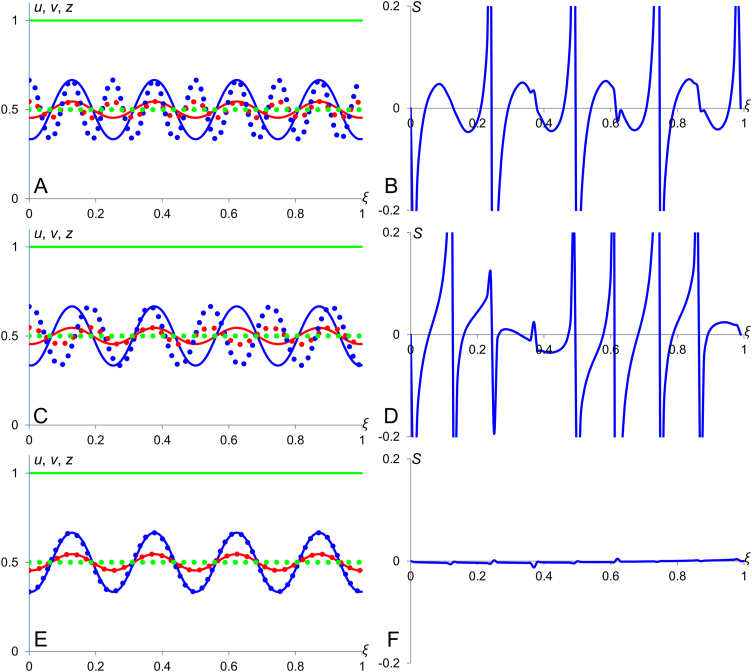
Scaling of Turing pattern in a system with modulation. Morphogen profiles and their sensitivity factors are shown for *n*=0, 1 and 2 in the system (19, 3). Panels A, C and E show the Turing patterns for *n*=0, 1 and 2 respectively. The continuous and dotted profiles stand for *L*=1 and *L*=2 respectively. The colours blue, red and green correspond to the profiles of *v* (activator), *z* (inhibitor) and *u* (modulator) respectively. Panels B, D and F show the sensitivity factors for profiles of *v* from panels A, C and E respectively. Values of model parameters: *D_u_*=1000, *D_v_*=1, *D_z_*=20, *k_z_*=1, *q*=0.001, *a*=0.2, *b*=−0.4, *c*=0.6, *d*=−0.8, *α_v_*=5.0 and *v*_0_=*z*_0_=0.5.

Here, *D*_*v*_ and *D*_*z*_ are the diffusion coefficients for *v* and *z* respectively and *a*, *b*, *c* and *d* are constants whose particular values allow formation of Turing patterns (Murray, 1982, Murray, 2003) and constant *α*_*v*_ is associated with an additional cubic term. For *n*=0, we have a system without modulator where the characteristic length of the Turing pattern does not depend on the size of the medium and therefore the number of spikes in the pattern is proportional to the medium size. For *n*=1 characteristic length of Turing pattern is increasing with the medium size but not quick enough to conserve the number of spikes. Finally, for *n*=2 we have a modulation resulting to perfect scaling when the characteristic length is proportional to the medium size and number of “spikes” in periodic pattern forming due to Turing instability does not depend on the medium size, *L.* This is illustrated by Fig. 6: there are four stripes in Turing pattern forming in the medium of size *L*=1 while when *L*=2, there are eight, five and a half and four stripes for *n*=0, 1 and 2 (in panels A, C and E) respectively. Sensitivity factor shown in panel F, which corresponds to *n*=2, is much smaller as compared to those shown in panels D and B which correspond to *n*=1 and *n*=0 respectively.

### 1.8 Scaling in activator-inhibitor systems without modulation

Although the number of spikes (stripes, spots, etc) in Turing pattern increases with the size of the medium, certain number of spikes can be observed in a range of medium sizes. Here, we aim to analyse scaling properties of Turing patterns when the medium size varies in a range such that the number of spikes does not change. We focus on a simple and probably the most common case of a single spike forming in activator–inhibitor systems (Meinhardt and Gierer, 2000). We compensate the luck of analytical tools available for this study by doing simulations using three different models. The first one is essentially the system (19) without modulation:

(20) $$\begin{array}{c} \frac{\partial v}{\partial t}=D_{v}\frac{\partial^{2}v}{\partial x^{2}}+\gamma_{v}\left(a\left(v-v_{0}\right)+b\left(z-z_{0}\right)-\alpha_{v}{\left(v-v_{0}\right)}^{3}\right)\\ \frac{\partial z}{\partial t}=D_{z}\frac{\partial^{2}z}{\partial x^{2}}+\gamma_{z}\left(c\left(v-v_{0}\right)+d\left(z-z_{0}\right)+\alpha_{z}{\left(z-z_{0}\right)}^{3}\right) \end{array}$$

Parameters *a*, *b*, *c* and *d* are assigned values such that so that the variable *v* acts as an activator, *z* as inhibitor and the homogeneous stationary state is unstable; constants *α*_*v*_ and *α*_*z*_, associated with additional cubic terms, define the amplitude of spikes; constants *γ*_*v*_ and *γ*_*z*_ allow variations in a number of unstable modes in the system (Maini et al., 2012) and assigned values such that a single spike can form in a large range of medium sizes.

The second model is the one introduced by Meinhardt and widely used by other researchers (Koch and Meinhardt, 1994):

(21) $$\begin{array}{c} \frac{\partial v}{\partial t}=D\frac{\partial^{2}v}{\partial x^{2}}+\frac{v^{2}}{z}-v+\sigma \\ \frac{\partial z}{\partial t}=\frac{\partial^{2}z}{\partial x^{2}}+k\left(v^{2}-z\right) \end{array}$$

This model is commonly considered in a range of parameters when the stationary homogeneous state is unstable (Koch and Meinhardt, 1994). The third model used in this study is the Fitzhugh–Nagumo (FHN):

(22) $$\begin{array}{c} \frac{\partial v}{\partial t}=D_{v}\frac{\partial^{2}v}{\partial x^{2}}-kv\left(v-1\right)\left(v-a\right)-z\\ \frac{\partial z}{\partial t}=D_{z}\frac{\partial^{2}z}{\partial x^{2}}+\varepsilon \left(lv-z\right) \end{array}$$

with a set of parameters corresponding to excitable dynamics (Vasiev, 2004). A spike in this model is initiated by applying a stimulus in the middle of the medium.

**Fig. 7:**
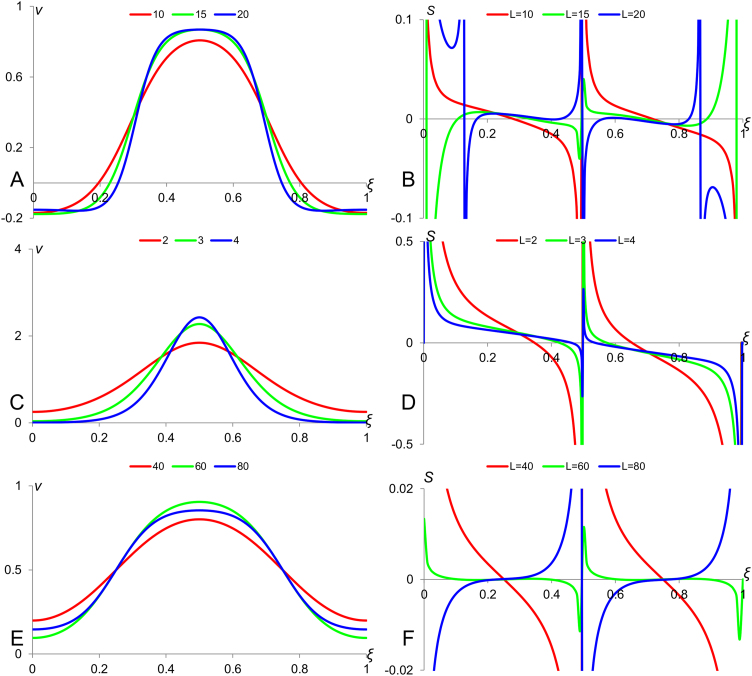
Scaling of a single spike forming in activator-inhibitor systems. Panels A, C and E show concentration profiles of activator obtained numerically for three different sizes of medium in FHN, Meinhardt and Turing models correspondently. Panels B, D and F show sensitivity factors for patterns shown on corresponding panels on the left. Model simulations were performed using explicit Euler method and central differencing scheme for diffusion terms. The parameters' values are as follow: For FHN model (Eq. (22)): *D_v_*=1, *D_z_*=20, *a*=0.05, *l*=1, *ε*=0.5, *k*=4 (Panels A and B). For Meinhardt model (Eq. (21)): *D*=0.05, *σ*=0. *k*=1.1 and up=0.5 (Panels C and D). For Turing model (Eq. (20)): *D*_v_=1, *D_z_*=20, *γ_v_*=0.04, *γ_z_*=0.02, *a*=1, *b*=−2, *c*=3, *d*=−4, *α_v_*=2.5 and *α_z_*=5 (Panels E and F).

Patterns forming in these three models under zero-flux boundary conditions have been found numerically. Single-spiked *v-*profiles and corresponding sensitivity factors are shown in Fig. 7. Panels A, C and E show patterns forming in the FHN, Meinhardt and Turing models respectively, while panels B, D and F contain plots of corresponding sensitivity factors. For each model *v-*profiles obtained for three different medium sizes are shown. We can see that the variations in the medium size result to the deformation of *v-*profiles (panels on the left side) and obtained sensitivity factors do not indicate reasonably good scaling (panels on the right side). The relatively convincing case of scaling is observed on the data obtained on Turing model. Namely, we can see that there are certain points in the medium *(ξ*≈0.25 and *ξ*≈0.75) where value of *v* (*v*≈0.5) does not change when the medium size is altered and were sensitivity factor stays close to zero for all considered medium sizes. Thus, if *v=*0.5 is a threshold value of concentration required for the differentiation then the relative position of the point with this concentration doesn’t change when the medium size is varied and therefore the differentiation pattern scales. Patterns forming in Meinhardt and FHN models do not have such points.

### 1.9 Scaling in two-dimensional activator-inhibitor systems

**Fig. 8:**
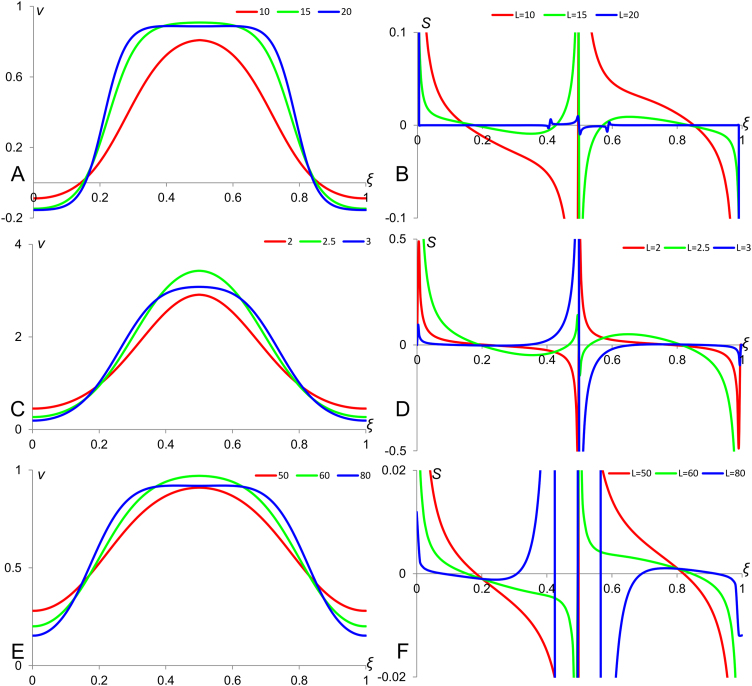
Scaling of a radially-symmetric spike forming in two-dimensional systems. Panels A, C and E show concentration profiles of activator obtained numerically for three different sizes of medium in FHN, Meinhardt and Turing models correspondently. Panels B, D and F show sensitivity factors for patterns shown on the corresponding panels on the left. Simulations were performed using explicit Euler method and central differencing scheme for diffusion terms. The parameters' values are the same as in Fig. 7.

So far, we have considered the problem of scaling for patterns forming in one-dimensional media. However, the most common biological setting for formation of morphogenetic pattern is rather two-dimensional, for example these patterns form in embryos which are commonly represented by epithelial (two-dimensional) tissues. Therefore, an important question to ask is how results obtained for 1D-systems change after transition to 2D-systems. This is a big and challenging problem which can be reduced to a number of smaller problems. For systems with passive modulation (like the one given by Eqs. (3), (9)), this question is answered by referring to physical dimensions of model parameters and variables (Ishihara and Kaneko, 2006). Another relatively simple case is represented by properties of radially-symmetric 2D-patterns which are simple enough to address mathematically and still important for biological applications. In this section, we focus on scaling properties of radially symmetric patterns forming in the considered above versions of Meinhardt, FHN and Turing models. Radially-symmetric patterns can be obtained in these models after small modification of all involved equations, namely when the diffusion term in Eqs. (20), (21), (22) (where the second derivative is now replaced by two-dimensional Laplacian) is represented in polar coordinates, that is:

(23) $$\frac{\partial^{2}v}{\partial x^{2}}+\frac{\partial^{2}v}{\partial y^{2}}=\frac{\partial^{2}v}{\partial r^{2}}+\frac{1}{r}\frac{\partial v}{\partial r}$$

when *r*=0 corresponds to the symmetry centre of the pattern. Patterns forming in these quasi-two-dimensional systems have been found numerically and shown in Fig. 8. Similarly to Fig. 7, Panels A, C and E show *v*-profiles obtained for three different medium sizes in FHN, Meinhardt and Turing models respectively. The shown radially-symmetric patterns are centred at *ξ*=0.5. Their sensitivity factor is represented in panels B, D and F respectively. Comparing plots on these panels with the ones shown on corresponding panels in Fig. 7, we can state that the scaling of patterns in Meinhardt model is improved (sensitivity factors in 2D-system are lower than in 1D-system) while in Turing model got worse. Besides, in the FHN model the sensitivity factor at points *ξ*≈0.15 and *ξ*≈0.85 where *v*=0 is close to zero and thus the differentiation pattern would scale well if the threshold for differentiation is given by *v*=0. Similarly, in the Meinhardt model the sensitivity factor at points *ξ*≈0.2 and *ξ*≈0.8 where *v*≈0.8 is close to zero making possible good scaling of differentiation patterns if the differentiation threshold is given by *v*=0.8.

## 2 Discussion

The problem of scaling of morphogenetic patterns has been addressed by many researchers and becomes increasingly attractive for mathematical studies in developmental biology (Ishihara and Kaneko, 2006, Lo et al., 2015, Umulis, 2009). Main focus of previous studies was on conditions allowing perfect patterning which is associated with the characteristic length being proportional to the medium size. (Umulis and Othmer, 2013). In this paper we have introduced a quantity, named “sensitivity factor”, which we used for the estimation of scaling quality of patterns forming in a few commonly considered systems and concluded on scaling properties of these patterns.

The first studied case is represented the diffusion-decay systems (when the characteristic length is given by the ratio $\lambda =\sqrt{D/k}$) for which we have shown that the scaling is always better under Dirichlet boundary conditions as compared to Neumann and perfect scaling is achieved only in the case of infinitely large characteristic length under Dirichlet boundary conditions. We have also shown that the variable described by the diffusion/decay equation with large characteristic length under Neumann boundary conditions levels off at a value dependant on the medium size and thus can be used to measure the medium size. Coupled properly to another variable representing the morphogen concentration it allows its scaling and thus can act as a modulator.

Our analysis of the systems with active transport indicates that adding advection to diffusion-decay systems does not lead to better scaling properties unless some special requirements are applied to the advection term. We have considered the advection in a model of active transportation of molecules along microtubules connecting the nuclei, so that the rate of advection is proportional to the concentration of nuclei and inverse proportional to the system's size if the number of nuclei in it is conserved. We have shown that strong advection in this model can result to better scaling of the morphogen profile.

We have also considered a case of cellular differentiation being regulated by two signals of which one is promoter and another – repressor. In this case scaling of the differentiation pattern does not directly correlate with the scaling of morphogenetic gradients. We have shown that in a simple case when the differentiation signal is a linear function of the promoter and repressor concentrations the scaling can be considerably improved in a model involving opposing gradients. Thus, models similar to the annihilation model (Ben-Naim, 1992) can be extremely helpful for analysis of differentiation patterns in biological tissues.

For our study of scaling of patterns forming in nonlinear systems, we have picked up a case of two-variable activator-inhibitor systems represented by three most commonly used models, namely modified FitzHugh-Nagumo (Vasiev, 2004), Meinhardt (Meinhardt and Gierer, 2000) and Turing (Murray, 1982) models. Using cubic version of Turing model we have illustrated that the passive modulation mechanism can preserve the number of spikes in Turing pattern forming in media of different sizes (Ishihara and Kaneko, 2006). Furthermore, we have numerically studied the scaling properties of Turing pattern represented by a single spike when no modulation is involved. Our simulations indicated that the scaling properties of Turing pattern when no modulator is involved are generally not good. Promising message is brought up only by some points in the medium where the sensitivity factor is close to zero in a wide range of medium sizes. This indicates that the patterns of differentiation in these models can scale if the concentration of activator in these particular points correspond to the threshold concentrations responsible for differentiation of cells (this kind of scaling mechanism was also considered by other authors (Umulis and Othmer, 2012)). Our analysis of radially symmetric patterns have shown that there is no direct correlation between scaling in one- and two-dimensional systems, and models showing good scaling in 1D are not the same which better scale in 2D.

The passive modulation mechanism of scaling proposed in this work is based on the observation that quickly diffusing/slowly decaying substance can level off over the biological tissue and its concentration can be used to measure the tissue size. Then, such substance can modulate the kinetics of another substance (morphogen) in a way that the latter's concentration profile is scaled. It is known that substances involved in patterning of biological objects typically have different kinetic/diffusion rates and, in some cases, there were identified proteins (i.e. Lefty in zebra fish embryo (Muller et al., 2012)) whose concentration doesn’t change very much over the tissue. In our models (see (9)), the morphogen *v* can reflect the Bicoid concentration profile while *u* can represent some hypothetical morphogen.

Our modelling results can be compared with biological observations, for example with experimental data on morphogen gradients in fly and zebra fish embryos (Gregor et al., 2008, Muller et al., 2012). This data indicate that the characteristic length of morphogen gradient is typically about a third of the medium size while the read-out of the morphogen concentration takes place in the middle third of the embryo. Furthermore, a four-fold increase in the size of the fly embryo results in less than 10% shift of the “mid-point” of the profile (Gregor et al., 2008), i.e. the point where the value of morphogen is equal to that in the middle (*ξ*=0.5) of small embryo remains in the range ±0.1 of the embryo size from the middle of the large embryo. This corresponds to *S*(*ξ*=0.5, *λ*/*L*=1/3)<0.03 which is at least by the order of magnitude smaller than sensitivity factors in the middle of the medium described by the diffusion-decay system with *λ*/*L*=1/3 for the either type of boundary conditions shown in Fig. 2. However the scaling of this quality can be obtained in other considered models including passive modulation (Eqs. (3), (9)), active transport (Eq. (14), with some additional assumptions) and annihilation model (Eq. (18)).

Biological illustration of our results on activator–inhibitor system can be given by the classical Lefty/Nodal system, where Nodal can be considered as an activator and Lefty – as inhibitor (Muller et al., 2012). It is known that the pattern of cellular differentiation does not change in a growing embryo indicating that the threshold signal responsible for the differentiation (for simplicity can be considered as the concentration of Nodal) is achieved at a point whose relative position does not change with an increase of the embryo. Scaling of the concentration profile of Nodal can also be explained by the modulation mechanism: Lefty represses Nodal, and this repression can reflect the fact that the decay rate of Nodal increases with the concentration of Lefty, bringing this to the model given by the Eqs. (3), (9).

Mechanism of passive modulation as applied to Turing patterns can explain formation of irregular patterns, such as those formed by gap genes in the fly embryo (Jaeger, 2009). Irregularities in these patterns represent a great challenge for understanding (Sanchez and Thieffry, 2003), mainly because they are so different from periodic Turing patterns. If we assume that the Turing pattern is modulated by a substance which has an exponential (rather than constant) profile then it is not going to be periodic anymore. Furthermore, if the modulator of the Turing pattern is also modulated, i.e. its concentration is defined by the system (3), (9) then the irregular pattern will be capable to scale too. These considerations open large field for further explorations of the properties of modulated morphogenetic patterns.

The modulation mechanism presented here can also be applied for analysis of patterns forming in dynamic systems such as those involving oscillations and propagating waves. Segmentation in vertebrate embryos, which is explained by the clock-and-wavefront mechanism (Cooke and Zeeman, 1976), results to scaled patterns. It is shown that this scaling is associated with the fact that phase shift for waves propagating through the segmentation region doesn’t depend on the size of this region (Lauschke et al., 2013). The preservation of the phase shift indicates that the velocity of waves crossing the tissue is proportional to the size of the tissue. This proportionality can be explained by modulation mechanism, i.e. the velocity is regulated by a concentration of modulator which, in turn, depends on the size of the tissue (Signon et al., 2016). Application of scaling mechanisms presented in this article to patterns forming in dynamical systems and to patterns in their transient phase (before reaching stationary state) (Bergmann et al., 2007) opens another large area for future research.
